# Communicating effectiveness of intervention for chronic diseases: what single format can replace comprehensive information?

**DOI:** 10.1186/1472-6947-8-25

**Published:** 2008-06-19

**Authors:** Henrik Stovring, Dorte Gyrd-Hansen, Ivar S Kristiansen, Jorgen Nexoe, Jesper B Nielsen

**Affiliations:** 1 Research Unit for General Practice, University of Southern Denmark, Odense, Denmark; 2 Institute of Public Health, University of Southern Denmark, Odense, Denmark; 3 Danish Institute of Health Services Research, Copenhagen, Copenhagen, Denmark; 4 Institute of Health Economics, University of Oslo, Oslo, Norway

## Abstract

**Background:**

There is uncertainty about how GPs should convey information about treatment effectiveness to their patients in the context of cardiovascular disease. Hence we study the concordance of decisions based on one of four single information formats for treatment effectiveness with subsequent decisions based on all four formats combined with a pictorial representation.

**Methods:**

A randomized study comprising 1,169 subjects aged 40–59 in Odense, Denmark. Subjects were randomized to receive information in terms of absolute risk reduction (ARR), relative risk reduction (RRR), number needed to treat (NNT), or prolongation of life (POL) without heart attack, and were asked whether they would consent to treatment. Subsequently the same information was conveyed with all four formats jointly accompanied by a pictorial presentation of treatment effectiveness. Again, subjects should consider consent to treatment.

**Results:**

After being informed about all four formats, 52%–79% of the respondents consented to treatment, depending on level of effectiveness and initial information format. Overall, ARR gave highest concordance, 94% (95% confidence interval (91%; 97%)) between initial and final decision, but ARR was not statistically superior to the other formats.

**Conclusion:**

Decisions based on ARR had the best concordance with decisions based on all four formats and pictorial representation, but the difference in concordance between the four formats was small, and it is unclear whether respondents fully understood the information they received.

## Background

Chronic disease processes (*e.g. *atherosclerosis, osteoporosis, carcinogenesis) account for 80–85% of all mortality in Norway and the UK (Statistics Norway, Statistics UK), and probably most industrialised countries. A considerable proportion of GPs' time is devoted to the detection and interventions related to such processes through case-finding, screening, life-style changes, pharmaceuticals or other medical interventions. In shared decision making GPs are supposed to inform patients about the effectiveness of such interventions [1]. This represents a considerable challenge to busy doctors who do not have the time to provide patients with comprehensive information about effectiveness of therapies. In practice, they may choose to provide brief information, and the question is how this is best done. While clinical trials quantify the health consequences of the interventions under ideal, controlled conditions, the effect is often diluted in realistic, non-controlled settings, where studies have found that only few interventions yield more than 12 months in additional average life time [2]. One likely explanation is that neither patients nor doctors experience any immediate effects, except for potential side effects, and therefore patients may lose the motivation for adhering to the treatment. This is frequently the case for statins and bisphosphonates [3], where interventions often are discontinued after 1–2 years. In part, this suboptimal adherence may be a consequence of the patient not feeling sufficiently well-informed about the potential benefits of adhering to treatment [4].

The standard, single formats of intervention effectiveness are: absolute risk reduction (ARR), relative risk reduction (RRR) and number needed to treat (NNT). In contrast to these formats that are measured at a specific point in time, prolongation of (disease-free) life (POL) has been suggested as a measure that summarises gain over the entire time scale. This has also been termed postponement of adverse outcomes [4]. None of these formats as such inform patients about their baseline risk, *i.e. *for example the heart attack risk during the subsequent 10 years.

Little is known about which single format, information should have to achieve decisions that most closely reflect the decision made when the patient has received comprehensive and nuanced information, and therefore at least in theory is in a better position to make qualified decisions. While others have shown that decisions can be manipulated by framing the information in a particular format [5], we are not aware of any studies that have focused on identifying which single format(s) that leads to decisions that would be upheld when given comprehensive information.

A further issue is the way in which each format is presented. ARR and RRR can be presented in terms of percentages, relative frequencies and pictorial representation. NNT can also be presented in different ways [5], and POL can be presented with or without information about the distribution around the mean value. While it is known that different presentations of essentially the same information influence consent rates [5,4,6], less is known about how this influences patients' ability to reach a decision they will maintain when given comprehensive information. As natural frequencies generally seem to be better understood than mere risk estimates [7], we included a pictorial representation of the expected treatment gain in the final, comprehensive information that respondents were presented with.

A priori, it would seem questionable that any one single format could capture the full range of interpretations of outcome data, as each format to some degree represents different aspects of the "truth". Estimates of prolongation of life without adverse events, whether they stem from clinical trials or simulation models, are estimates of the average or expected postponement of the adverse event. In practice, it is impossible to infer the size of the prolongation for the individual patient because small prolongations in most or all patients may create the same survival curves as large prolongations in a few. In other words, we can infer the average health gain, but not its distribution among those having therapy. It is therefore impossible to determine whether the effect is "small for all" or a few are winning "the big prize" from empirical data [8]. Although ARR and average prolongation are generated from the same data, the two formats "invite" the decision maker to interpret them differently: ARR signals that a big prize (health benefit) is won by the few, while average prolongation signals smaller gain for all. Whatever the true distribution would be in a given situation, it is important to investigate which single format leads to the same decisions as those based on comprehensive information.

The aim of this study was to explore which single format results in decisions that are closest to decisions made by the same individual when given comprehensive information. It is important to emphasize that comprehensive information is not equivalent of unbiased, perfect information – it is in this context a collective presentation of the various formats (ARR, NNT, RRR and POL) including a pictorial representation. If decisions made on the basis of one single, specific interpretation of the data, *i.e. *one format, differ significantly from the decisions made when the more nuanced story is told, this will indicate that this single format cannot necessarily replace comprehensive information. Because it is difficult to obtain a sufficiently large number of patients for such a study in a clinical setting, we carried out a randomized study with interviews of a representative sample of the general population.

## Methods

In the autumn of 2005, a representative sample of individuals aged 40–59 in the municipality of Odense, Denmark, (approximately 185,000 inhabitants) was invited for an interview. Interviews took place in a university building next to the main hospital just outside the city centre. The individuals were informed that interviews would be about preventive healthcare and would take about 10–15 minutes. For their efforts, the respondents would receive either two bottles of wine or one box of luxury chocolate. In total, 4,000 individuals were randomly selected among people aged 40–59 in Odense as of January 1, 2005, and 1,491 (37%) were successfully interviewed. Interviews took place during four-hour sessions in the afternoon over a period of six weeks. Non-responders were followed up with reminders by letter and telephone. All interviews were performed by professional and trained interviewers from The Danish National Centre for Social Research.

The design of the interview guides were the result of lengthy discussions within the research group, three different focus groups, and ultimately with researchers from the The Danish National Centre for Social Research that performed the interviews. In this paper, we present results for 16 of the 24 interview guides. Here, the respondents were first asked about age, gender, marital status, family income, educational attainment and occupation. Subsequently, they were asked to imagine that they were at an increased risk of a heart attack and offered a pharmaceutical drug. No drug name was mentioned, but data on effectiveness and side effects resemble the outcomes associated with statins. The respondent then received information on treatment effectiveness in terms of one single information format, and subsequently in terms of a picture in combination with data on NNT, RRR and life extension (POL) (Appendix1 [see Additional file 1]). After each round of information, the respondents were asked whether they would consent to therapy (yes, no, uncertain) and further to indicate the strength of their consent on a scale from 1 (="not at all") to 10 (="to a very high degree"). Then, respondents were asked which one of the four information formats they liked most, how difficult it was to understand this preferred format, whether they or their closest relative had hypercholesterolaemia, hypertension, or had had a heart attack or stroke. At the end they were asked four questions that capture numeracy (one taken from an existing questionnaire [9,10], and three questions made by the authors). All information was read to the respondents while they simultaneously could read the information on cards (cf. Appendix 2, where the cards associated with the single information ARR is presented as an example. Other formats had the initial information replaced by the appropriate format present on the card with comprehensive information [see Additional file 2]). When the interview was finished, the interviewers rated their perception of the respondents' understanding of card 3 (the "comprehensive information"). This rating was based on the interviewer's subjective impression of understanding. The full interview guide is presented in Appendix 3 [see Additional file 3].

We aimed to test concordance for different levels of baseline risk and effectiveness in order to explore whether the results were specific for specific levels of risk or effectiveness. We used 5% and 15% 10-year risk of a fatal heart attack because these are realistic levels for situations where the GP would consider pharmaceutical treatment. We used 33% for RRR because this is a usual level for statin treatment, while we also assigned RRR of 40%, 66% and 80% (Table 1). These additional RRR levels were chosen to fit the study aims of formats 17–24.

**Table 1:** Study design: The 16 interview formats according to baseline risk of heart attack, level of effectiveness, and initial piece of information

		ARR		RRR		NNT		POL	
Baseline Risk	Level of effectiveness	Effect size	n	Effect size	n	Effect size	n	Effect size	n
Low (5%)	Low	2%	79	40%	79	50	68	4 mths	69
	High	4%	75	80%	69	25	69	8 mths	67

High (15%)	Low	5%	71	33%	73	20	82	8 mths	86
	High	10%	69	66%	71	10	69	16 mths	73

Total			294		292		288		295

Consequently, we had 4 different risk level and effectiveness groups. Combined with baseline risks, corresponding effect estimates could be derived for the three other risk formats (derivations not shown, but available upon request). For each set of baseline risk and effect size, either ARR, RRR, NNT or POL was used as the initial single information piece and thus 16 different interview formats were used (Table 1). Additionally, we had eight other interview guides with discrete choice questions that are not included in the present analysis.

Because there were 24 different interview guides, each with a unique set of information cards, we chose to let each interviewer have one interview guide with the associated cards during each interview session (afternoon). This was first to avoid mismatch between the format registered for each respondent, the cards and the interview guides, and second to keep the number of printed guides and format at a reasonable level. No single interviewer did more than 37 interviews with any given guide of the 16 available, and all guides had at least 5 interviewers associated with them. The respondents were assigned one of the 24 guides according to the sequence of attending: The sequence of the guides was random. Even though this design does not represent perfect randomisation, we considered it to represent the best trade-off between randomness, feasibility and avoidance of mismatch between registered and actual interview format.

The study was powered to detect a 15% difference in proportion consent between two guides. We consequently aimed to have 100 respondents for each interview guide, in total 2,400 interviews.

## Statistics

The basic response variable was consent to treatment after the initial and final information, measured both on a binary and a 10-point Likert scale. From the binary responses we computed concordance as an indicator of whether or not the initial choice was upheld in the final decision. From the Likert scale responses we computed the difference between final and initial score, *i.e. *a negative difference indicates that the subject became less willing to accept treatment. As explanatory variables we used the type of initial format (ARR, RRR, NNT, POL), level of baseline risk (high, low), size of treatment effectiveness (high, low), age (40–44 yrs, 45–49 yrs, 50–54 yrs, 55–60 yrs), gender, cohabitant or not, less than two correct responses to the four numeracy questions (yes, no), personal experience with cardiovascular disease (yes, no), experience with cardiovascular disease in family (yes, no), and the interviewer's assessment of whether the informant seemed to understand the presented information (yes, no).

To account for non-response to individual items, we employed multiple imputation using the so-called *ice *and *micombine *procedures available in Stata 9.2 [11,12]. In the imputation step we used all available information for all variables described above to generate the ten completed datasets. Subsequently the data were analysed using either logistic regression for binary responses (consent and concordance: yes/no), or ordinary linear regression for Likert scale outcomes (rated preference and difference in rated preference). For the latter outcomes we used robust variance estimates to account for departures from normality [13,14]. For all estimates we report 95% confidence intervals in parentheses.

## Results

Table 1 shows the design of the experiment and the number of respondents in each of the sixteen groups. While the overall participation rate was low (n = 1491, 37.3%), the randomisation was successful in creating groups of equal size and composition. Compared to the background population, the participants were older (median age 51 among participants, 49 among non-participants) and a higher proportion were women (57.1% among participants, 45.2% among non-participants). Further, the participants had a lower mean annual household income of DKK 507 k, where the average for 40 to 49 year-olds was DKK 639 k and for 50–59 year-olds DKK 600 k in the general Danish population. Finally, participants had a longer education than the general population in this age group: Only 11% had a shorter education than 9 years among participants, whereas this is 28% in the general population. Furthermore, 24% of participants had an education longer than 18 years, whereas this was the case for 7% in the general Danish population in this age group. From now on we only report on the 1169 subjects randomised into the 16 groups relevant for this study.

Table 2 shows the characteristics of the informants according to the initial type of information they were presented with. While there was a slight variation between the groups, the randomisation seems to have been successful in creating groups with similar characteristics among respondents. Response rates for individual items among respondents were generally very high.

**Table 2:** Descriptive statistics of the respondents according to interview format

	ARR		RRR		NNT		POL	
Covariate	n	p/median	n	p/median	n	p/median	n	p/median
Sex (Females, %)	294	56.5	292	54.8	288	53.5	295	61.0
Age (years)	294	52 (43; 59)	288	51 (43;59)	288	51 (42;59)	295	51 (42; 59)
Household income (1,000 DKK)	292	500–599 (100–199; 900–999)	289	500–599 (100–199; 800–899)	281	500–599 (200–299; 1,000+)	293	500–599 (200–299; 800–899)
Education (years)	294	13 (10; 17)	292	13 (9; 16)	285	13 (10; 16)	290	12 (9; 17)
Married or cohabitating w/partner (Yes, %)	294	73.8	292	74.0	287	81.2	293	78.5
Personal experience w/cardiovascular disease (Yes, %)	292	39.4	290	36.2	287	33.1	295	38.3
Family w/known cardiovascular disease (Yes, %)	287	41.1	281	36.3	286	42.3	288	45.5
Numeracy skills (2 or more wrong answers, %)	294	19.7	292	18.2	288	24.0	295	17.6

For proportions, percentages are given, while for continuous and categorical variables the median is given with 10% and 90% percentiles in parentheses. n indicates the number of valid responses

In Table 3 we present proportions of respondents willing to accept treatment after the initial and final information, and the corresponding concordances, stratified by format, level of baseline risk, and level of effectiveness. Concordance is finally displayed stratified according to initial consent status with respect to therapy. Similarly, in Table 4 we present the average scores on the Likert scale after initial and final information, as well as the average of individual differences. In general, the proportion of informants accepting treatment was about 70% regardless of initially presented outcome format. In fact, across all formats and both rounds of questions, the lowest proportion was 53% and the highest 78% (Table 3). The subjects were somewhat sensitive to the level of effectiveness in terms of their willingness to accept treatment, be it binary or on the Likert scale, but the picture was not entirely consistent. An entirely consistent pattern would imply that the proportions consenting to therapy increased monotonously with increasing effectiveness, and that was not the case (see Table 3). This pattern was repeated for concordance, but not for individual differences on the Likert scale, where the level of effectiveness seemed to play a less important role. The format resulting in the highest concordance was ARR, followed by RRR, POL, and NNT, in that order, but differences were small. The smallest average difference was achieved with the ARR and NNT formats jointly, followed by RRR and POL, in that order. Again the differences in movements on the Likert scale across formats were small.

**Table 3:** Preferences for therapy: proportions of respondents consenting to the proposed therapy with binary response (yes/no)

		Proportions (%) consenting to initial proposal			
Baseline Risk	Level of effectiveness	ARR	RRR	NNT	POL
Low	Small	60 (48; 70)	65 (53; 75)	56 (43; 68)	49 (37; 61)
	Large	71 (59; 80)	78 (65; 87)	67 (54; 77)	59 (47; 70)

High	Small	72 (60; 81)	77 (65; 85)	70 (59; 80)	53 (43, 64)
	Large	67 (55; 77)	73 (61; 82)	73 (61; 83)	64 (53; 74)

Total		67 (61; 72)	73 (67; 78)	67 (61; 72)	56 (51; 62)

		Proportions (%) consenting to final proposal			
Baseline Risk	Level of effectiveness	ARR	RRR	NNT	POL

Low	Small	59 (48; 70)	57 (46; 68)	52 (40; 64)	60 (47; 71)
	Big	71 (59; 80)	71 (59; 81)	64 (51; 74)	66 (54; 76)

High	Small	70 (58; 80)	69 (57; 79)	71 (60; 80)	60 (49; 70)
	Big	67 (55; 77)	73 (61; 82)	79 (68; 87)	71 (60; 80)

Total		67 (61; 72)	67 (62; 73)	67 (61; 72)	64 (58; 69)

		Concordance (%) between initial and final response			
Baseline Risk	Level of effectiveness	ARR	RRR	NNT	POL

Low	Small	90 (81; 95)	93 (83; 97)	84 (72; 91)	86 (76; 93)
	Big	100 (94; 100)	91 (80; 96)	91 (81; 96)	93 (83; 97)

High	Small	92 (82; 97)	88 (78; 94)	91 (82; 96)	90 (81; 95)
	Big	97 (89; 99)	94 (85; 98)	90 (79; 95)	93 (85; 97)

Total		94 (91; 97)	91 (87; 94)	89 (84; 92)	90 (86; 93)

		Concordance (%) between initial and final response according to initial response			
Initial consent?		ARR	RRR	NNT	POL

No		92 (84; 96)	94 (84; 98)	83 (74; 90)	80 (72; 86)
Yes		95 (91; 98)	90 (84; 94)	92 (86; 95)	98 (94; 100)

**Table 4:** Preferences for therapy according to interview format, Likert scale response

		Initial agreement to receive treatment on Likert scale			
Baseline risk	Level of effectiveness	ARR	RRR	NNT	POL
Low	Small	7.29 (6.65; 7.93)	6.16 (5.40; 6.91)	5.29 (4.44; 6.15)	5.39 (4.70; 6.08)
	Large	7.03 (6.31; 7.76)	6.64 (5.93; 7.34)	6.70 (5.94; 7.45)	6.13 (5.39; 6.88)

High	Small	6.03 (5.25; 6.81)	6.73 (6.05; 7.42)	6.63 (6.00; 7.26)	5.48 (4.82; 6.13)
	Large	6.36 (5.57; 7.15)	6.87 (6.18; 7.57)	6.87 (6.13; 7.61)	6.68 (5.97; 7.40)

Total		6.70 (6.33; 7.07)	6.59 (6.23; 6.95)	6.39 (6.01; 6.77)	5.91 (5.55; 6.26)

		Final agreement to receive treatment on Likert scale			
Baseline risk	Level of effectiveness	ARR	RRR	NNT	POL

Low	Small	6.97 (6.29; 7.66)	5.54 (4.75; 6.32)	5.00 (4.15; 5.86)	5.99 (5.26; 6.71)
	Large	6.88 (6.13; 7.63)	6.42 (5.68; 7.16)	6.55 (5.79; 7.31)	6.66 (5.92; 7.39)

High	Small	6.07 (5.32; 6.82)	6.29 (5.53; 7.05)	6.67 (6.03; 7.32)	5.94 (5.26; 6.62)
	Large	6.22 (5.44; 7.01)	6.54 (5.86; 7.21)	6.64 (5.92; 7.35)	6.86 (6.17; 7.56)

Total		6.56 (6.18; 6.93)	6.18 (5.80; 6.55)	6.24 (5.86; 6.62)	6.34 (5.99; 6.70)

		Difference between initial and final agreement to receive treatment on Likert scale			
Baseline risk	Level of effectiveness	ARR	RRR	NNT	POL

Low	Small	-0.32 (-0.70; 0.07)	-0.62 (-0.98; -0.26)	-0.29 (-0.79; 0.21)	0.59 (0.15; 1.04)
	Large	-0.16 (-0.48; 0.17)	-0.22 (-0.60; 0.17)	-0.15 (-0.50; 0.21)	0.52 (0.18; 0.87)

High	Small	0.04 (-0.19; 0.28)	-0.45 (-0.86; -0.03)	0.04 (-0.37; 0.44)	0.47 (0.04; 0.89)
	Large	-0.14 (-0.36; 0.08)	-0.34 (-0.70; 0.02)	-0.23 (-0.72; 0.26)	0.18 (-0.08; 0.44)

Total		-0.15 (-0.30; 0.01)	-0.41 (-0.60; -0.22)	-0.15 (-0.37; 0.07)	0.44 (0.25; 0.63)

		Difference between initial and final agreement to receive treatment on Likert scale with respect to initial choice			
Initial treatment acceptance		ARR	RRR	NNT	POL

No		0.10 (-0.12; 0.32)	0.03 (-0.28; 0.33)	0.52 (0.17; 0.87)	0.97 (0.64; 1.31)
Yes		-0.27 (-0.47; -0.07)	-0.58 (-0.82; -0.33)	-0.48 (-0.75; -0.21)	0.02 (-0.18; 0.22)

The last sub-tables of Table 3 and 4 do, however, reveal an additional pattern. Informants were generally most concordant when they initially accepted treatment, but with substantial differences between formats: For RRR, concordance was highest among those who initially declined treatment ("too many" consented to treatment initially compared to their final choice), for POL this is reversed ("too many" declined treatment initially compared to their final choice). For NNT, concordance was low regardless of the initial decision, while it was high for ARR, again regardless of initial choice.

In regression analyses of concordance and difference on Likert scales (Table 5) no statistically significant interactions between initial format and the other covariates were found. Most effects were small and not statistically significant, except for the association between level of effectiveness and concordance. None of the explanatory variables changed substantially when adjusted for the remaining covariates, *i.e. *we could not identify any important factors that could have confounded the overall results. This includes individuals' disease history, which only affected the overall tendency to consent to treatment, but not concordance. Hence, the format leading to the most concordant decisions according to the adjusted logistic regression was again the ARR followed by RRR, POL, and NNT. For the average difference on the Likert scale, the adjusted linear regression identified the sequence of optimal formats as ARR followed by NNT, RRR, and POL.

**Table 5:** Association between concordance or difference of initial and final treatment acceptance and format initially presented. All estimates are based on multiple imputation.

		Concordance		Difference	
		OR (Crude)	OR (Adjusted*)	Beta (Crude)	Beta (Adjusted*)
Initial format	ARR	1	1	(Ref)	(Ref)
	RRR	0.64 (0.33; 1.25)	0.62 (0.31; 1.24)	-0.41 (-0.60; -0.22)	-0.29 (-0.57; -0.02)
	NNT	0.48 (0.25; 0.92)	0.45 (0.23; 0.87)	-0.15 (-0.34; 0.04)	0.01 (-0.28; 0.29)
	Delay	0.57 (0.29; 1.11)	0.52 (0.26; 1.03)	0.44 (0.25; 0.63)	0.56 (0.28; 0.84)

Base line risk	Low	1	1	(Ref)	(Ref)
	High	1.10 (0.71; 1.71)	1.07 (0.68; 1.67)	0.06 (-0.14; 0.25)	0.03 (-0.16; 0.23)

Effect size	Low	1	1	(Ref)	(Ref)
	High	1.74 (1.12; 2.71)	1.76 (1.12; 2.77)	-0.00 (-0.20; 0.19)	0.01 (-0.19; 0.21)

* Adjustment was made for age, gender, living with a partner, numeracy, personal experience with cardiovascular disease, experience with cardiovascular disease in the family, and interviewer's assessment of whether the informant understood the information given.

## Discussion

Of the four formats used for initial information (ARR, RRR, NNT, POL), the ARR format led to the "best" decision in the sense that the decisions were upheld to a greater extent with ARR than for the other formats, but ARR was not statistically superior to the other formats.

The subjects were given incentives to improve participation rates, but it remained low at 37.3%. Further, participation rates were related to gender, age, and socio-economic status. While this may question the generalisability of the study, the internal validity would appear to be intact, as the randomisation was successful in creating equal sized and comparable groups, and as there were no drop-outs after randomisation. To further avoid drop-outs occurring as a side effect of incomplete responses – often only complete cases are included in statistical analyses – we employed the technique of multiple imputation to make use of all available information. Additional regression analyses without multiple imputations and consequently fewer respondents yield similar results, but with less precision (data not shown).

Although the study was intended to mimic a "real" treatment decision as much as possible, the study did depart from this ideal in that participants were not patients. We hence controlled for personal and/or familial experience of disease in analyses. While experience with disease did increase overall willingness to consent to therapy, it did not affect concordance significantly. In terms of identifying the "best" format, *i.e. *the one with highest concordance, our results can thus reasonably be expected to translate well into a clinical setting.

Participants were presented with rather large variations in the levels of treatment effectiveness, and yet the proportions of consent to therapy varied little. One may hypothesize two explanations for this insensitivity: (i) respondents do not understand the information they receive; (ii) respondents understand the information, but make decision on factors other than effectiveness. Several studies have shown that lay people are insensitive to levels of effectiveness in hypothetical treatment decision when the effectiveness is presented in terms of NNT [15-17]. The explanation may be evaluability heuristics created by the fact that lay people have little or no experience with evaluating NNTs. Without experience in evaluating effectiveness information, people may make decisions on the basis of factors that they understand or that create affect^15^. While levels of effectiveness do not seem to influence decisions when presented in terms of NNT, the cost of the treatment, the type of side effects and the type of disease have considerable influence in similar experiments [16]. Such aspects may be much easier to evaluate. While previous research indicates that NNT and RRR may be subject to evaluability heuristics, POL seems to be less so. In two experiments, lay people have been able to discriminate between levels of effectiveness when presented with a prolongation of life without adverse events [4,18]. Interestingly, even respondents who were presented with POL, were relatively insensitive to levels of effectiveness in the present study.

The relative insensitivity to level of effectiveness will tend to hide the influence of information format because most consent rates were in the range 60%–75%. The concordance proportions were generally high and this limited the scope for differences between formats. While this raises the question of the study not being sufficiently sensitive to detect differences in concordance proportions, it more importantly also raises the general question of whether or not subjects are capable of meaningful risk assessment and evaluation of the true benefit associated with a given treatment.

Even so, we did find significant differences between formats, in particular we identified clear directions of changes for two formats. Subjects initially presented with RRR generally became less likely to consent to treatment after receiving comprehensive information, while subjects initially presented with POL became more willing to accept treatment after having been given the fuller picture. These movements were most visible on the more sensitive Likert scale, than on the binary scale (yes/no). RRR has previously been reported to convey an overly optimistic impression on treatment efficacy [19] and the present observations support this in the sense that subjects initially given RRR information tend to adjust consent to treatment downwards when given information in other formats. For those initially presented with NNT information, the movements were rather large and bidirectional. The difference was +0.52 on the Likert scale for those who initially rejected the therapy while it was -0.48 for those who initially accepted it. The results may possibly reflect a lack of understanding of the NNT format as indicated in previous studies [18,15]. Only for ARR, changes in decision were infrequent and bidirectional. This may indicate that ARR accompanied by baseline risk information is closest to the pictorial representation of natural frequencies.

In the present study we use comprehensive information as a common benchmark for valuing the performance of the single formats. We argue that to the extent that single formats and comprehensive information produce the same results, one can reduce GP time and effort by providing patients with the single information format which concords best with comprehensive information. In drawing this conclusion, we do not infer that either of the two formats necessarily lead to optimal decisions. Information can only lead to optimal decision making if individuals understand and use all provided information in order to optimize their individual utility function. Clearly, we cannot verify whether this is the case. One may, however, argue that the comprehensive information respondents are provided with in the present study with a high probability should lead to more informed and thus *better *decisions than decisions made on the basis of single formats – because comprehensive information provides the patient with a nuanced and relevant array of information. As we have emphasized earlier, comprehensive information presents information on effectiveness in different formats, which reflect various possible distributions of outcomes – whereas the single format implies a single distribution, which is not necessarily the true distribution. Comprehensive information also includes pictorial representation of natural frequencies, which has been shown to be readily understood by many. Finally, comprehensive information in this study includes an explicit presentation of information on RRR. While one can argue that focus on RRR may bias decisions and lead individuals away from the relevant outcome (the gain in life-expectancy), it is a fact that RRR is a piece of information that is available as long as we provide patient with information on base-line risk *and *risk reduction. What our results have shown is that individuals presented with ARR and comprehensive information (including an explicit presentation of RRR) demonstrate high concordance which suggests that explicit presentation of RRR does not have a marked impact when this information is provided along with information on effectiveness.

To the best of our knowledge, no similar study has previously been performed. Some aspects of the study, however, have been elucidated in previous studies. Several studies have compared the consent rates for equal effectiveness with information formats [17,4]. Typically, respondents are more positive towards treatment when presented with RRR than with ARR, NNT or POL. Our results confirm this although we found relatively small differences across the four formats. The explanation may be that all respondents were informed about baseline risk which would tend to put RRR in perspective. Another explanation may be that the respondents suffered information overload and decided on the basis of factors other than effectiveness. Since the price of the treatment and the side effects were identical in the 16 interview formats, the consent rate may be relatively similar if respondents make decisions on the basis of such factors as price or side effects.

Because this is the first study to test the influence of four information formats and four effectiveness levels in the same study, one should interpret the results cautiously. The findings may suggest that RRR and POL can be used to manipulate decision makers because decisions made on one single of these formats seem to produce "optimistic" or "pessimistic" decisions. On the other hand it is not clear whether lay people who are informed about NNT or ARR make good decisions, nor is it clear whether effectiveness is a crucial issue for people who make treatment decisions. It is conceivable that people consider the severity of the potential disease, the treatment costs and side effects and put less emphasis on the magnitude of the effect as far as it is above some threshold which may be close to zero for some people.

The findings of this study and several others suggest that people make decisions not only on the basis of health outcome, but also on the basis of other factors such as price, convenience of care, etc. This study may indicate that we need just as much knowledge about how patients value health and non-health outcomes as about how to convey information about probabilities and effect sizes.

## Conclusion

While we conclude that ARR may represent the best single information format, the study raises questions about lay people's understanding even when special efforts are made to convey information in an understandable way. The study may also indicate that level of effectiveness is not a crucial issue when making a decision about preventive interventions.

## Competing interests

The authors declare that they have no competing interests.

## Authors' contributions

HS drafted and revised the manuscript, planned and conducted the statistical analyses. DG–H, ISK, JN, and JBN jointly conceived the idea of the study, designed the trial, and oversaw its implementation. All authors read and approved the final manuscript.

## Pre-publication history

The pre-publication history for this paper can be accessed here:



## Supplementary Material

Additional file 1 Outline of Information Cards. Five cards with illustrations and questions presented to interviewed subjects with initial information on ARR and subsequent comprehensive information. Click here for file

Additional file 2 All Information Cards for a single interview. All eight cards with illustrations and questions presented to interviewed subjects with initial information on ARR and subsequent comprehensive information. Click here for file

Additional file 3 Complete interview guide. Example of interview guide including text read by the interviewer, all 25 questions asked to respondents with possible responses, and an additional question to interviewers for evaluating the respondent's apparent understanding. Click here for file
