# Supplementary material for: Communicating effectiveness of intervention for chronic diseases: what single format can replace comprehensive information?
Source: BMC Med Inform Decis Mak. 2008 Jun 19;8:25. doi: 10.1186/1472-6947-8-25 (PMC2467410; doi:10.1186/1472-6947-8-25)
Supplement: Additional file 1 — Outline of Information Cards. Five cards with illustrations and questions presented to interviewed subjects with initial information on ARR and subsequent comprehensive information. [file 1472-6947-8-25-S1.doc]

# CARD 1

#### CARD 1A (INITIAL INFORMATION)

**ARR** (example): If 100 individuals like yourself takes the medicine, will 5 more be alive after 10 years, than if they do not take the medicine.

**Question:** Would you be willing to take the medicine:

o Yes

o No

o Uncertain

# CARD 2

If you rather had to respond on a scale, to what extent would you take the medicine? You can respond by means of this card where zero means not at all and 10 means to great extent.

| Not at all | 1 |
| --- | --- |
|  | 2 |
|  | 3 |
|  | 4 |
|  | 5 |
|  | 6 |
|  | 7 |
|  | 8 |
|  | 9 |
| To great extent | 10 |

# CARD 3 AND 4 (COMPREHENSIVE

**INFORMATION)**

### Without treatment With treatment

☹☹☹☹☹☹☹☹☹☹☻☻☻☻☻☻☻☻☻☻☻☻☻☻☻☻☻☻☻☻☻☻☻☻☻☻☻☻☻☻☻☻☻☻☻☻☻☻☻☻☻☻☻☻☻☻☻☻☻☻☻☻☻☻☻☻☻☻☻☻☻☻☻☻☻☻☻☻☻☻☻☻☻☻☻☻☻☻☻☻☻☻☻☻☻☻☻☻☻☻

☹☹☹☹☹☹☹☹☹☹

☹☹☹☹☹☻☻☻☻☻

☻☻☻☻☻☻☻☻☻☻

☻☻☻☻☻☻☻☻☻☻☻☻☻☻☻☻☻☻☻☻☻☻☻☻☻☻☻☻☻☻☻☻☻☻☻☻☻☻☻☻☻☻☻☻☻☻☻☻☻☻☻☻☻☻☻☻☻☻☻☻

☻☻☻☻☻☻☻☻☻☻

1. If 20 persons with elevated cholesterol level like yourself, take the medicine, one more person will be alive after 10 years, than if they do not take the medicine **(NNT)**
2. If a person with elevated cholesterol level like yourself, takes the the medicine, the risk of dying from heart attack will be reduced by 33% the next 10 years **(RRR)**
3. If persons with elevated cholesterol levels like yourself, takes the medicine, on average they will live for 8 months longer than if they do not take the medicine **(POL)**

**CARD 5**

Same questions as card 1A and 2
