# Supplementary material for: Communicating effectiveness of intervention for chronic diseases: what single format can replace comprehensive information?
Source: BMC Med Inform Decis Mak. 2008 Jun 19;8:25. doi: 10.1186/1472-6947-8-25 (PMC2467410; doi:10.1186/1472-6947-8-25)
Supplement: Additional file 2 — All Information Cards for a single interview. All eight cards with illustrations and questions presented to interviewed subjects with initial information on ARR and subsequent comprehensive information. [file 1472-6947-8-25-S2.doc]

# Card for No. 1

# CARD 1

Study no.: 5777

#### CARD 1.A

If 100 persons with raised cholesterol levels like you take the medication, 5 more would be alive after 10 years, than if they did not take the medicine

Study no.: 5777

## CARD 2

| Not at all | 1 |
| --- | --- |
|  | 2 |
|  | 3 |
|  | 4 |
|  | 5 |
|  | 6 |
|  | 7 |
|  | 8 |
|  | 9 |
| To a very high degree | 10 |

# Study no.: 5777

# CARD 3

### Without treatment With treatment

☹☹☹☹☹☹☹☹☹☹☻☻☻☻☻☻☻☻☻☻☻☻☻☻☻☻☻☻☻☻☻☻☻☻☻☻☻☻☻☻☻☻☻☻☻☻☻☻☻☻☻☻☻☻☻☻☻☻☻☻☻☻☻☻☻☻☻☻☻☻☻☻☻☻☻☻☻☻☻☻☻☻☻☻☻☻☻☻☻☻☻☻☻☻☻☻☻☻☻☻

☹☹☹☹☹☹☹☹☹☹

☹☹☹☹☹☻☻☻☻☻

☻☻☻☻☻☻☻☻☻☻

☻☻☻☻☻☻☻☻☻☻☻☻☻☻☻☻☻☻☻☻☻☻☻☻☻☻☻☻☻☻☻☻☻☻☻☻☻☻☻☻☻☻☻☻☻☻☻☻☻☻☻☻☻☻☻☻☻☻☻☻

☻☻☻☻☻☻☻☻☻☻

Study no.: 5777

# CARD 4

1. If 20 persons with raised cholesterol levels like you take the medication, one more will be alive after 10 years, than if they did not take the medication
2. If a person with raised cholesterol levels like you take the medication, the risk of dying of a coronary thrombosis will be reduced by 33% within the next 10 years
3. If persons with raised cholesterol levels like you take the medication, they will on average live 8 months longer, than if they did not take the medication

Study no.: 5777

## CARD 5

| Not at all | 1 |
| --- | --- |
|  | 2 |
|  | 3 |
|  | 4 |
|  | 5 |
|  | 6 |
|  | 7 |
|  | 8 |
|  | 9 |
| To a very high degree | 10 |

# Study no.: 5777

# CARD 7

1. Considerations for my health
2. I trust that the doctor knows what is best for me
3. I have a responsibility towards my family/loved ones to stay healthy
4. Anything else, please explain

Study no.: 5777

# CARD 8

1. I feel that the effect of the medication is too small
2. I wish to avoid the side-effects of the medication
3. I do not want to pay the extra expense
4. I do not like to take medication
5. I found it difficult to understand the information I received
6. I do not believe that the medication works
7. It is better to change my lifestyle than to take medication
8. Anything else, please explain

Study no.: 5777
