# Supplementary material for: Communicating effectiveness of intervention for chronic diseases: what single format can replace comprehensive information?
Source: BMC Med Inform Decis Mak. 2008 Jun 19;8:25. doi: 10.1186/1472-6947-8-25 (PMC2467410; doi:10.1186/1472-6947-8-25)
Supplement: Additional file 3 — Complete interview guide. Example of interview guide including text read by the interviewer, all 25 questions asked to respondents with possible responses, and an additional question to interviewers for evaluating the respondent's apparent understanding. [file 1472-6947-8-25-S3.doc]

**no.1**

**Information on preventive medicine**

Study no. 5777.

September - October

2005

| Text 1:  As you will see from the letter we sent you, the purpose of this study is to find out how to inform about the effect of preventive medicine in the best possible way |
| --- |

But first I have some questions about yourself

Question 1

How old are you?: ________ years

*(Interviewer: make a note of the age)*

Question 2

*(Interviewer: make a note of gender)*

1 Man

2 Woman

Question 3

What is your household’s total annual income - gross – i.e. before tax? *(only one tick)*

1. Under 100,000

2. 100,000 - 199,999

3. 200,000 – 299,999

4. 300,000 – 399,999

5. 400,000 – 499,999

6. 500,000 – 599,999

7. 600,000 – 699,999

8. 700,000 – 799,999

9. 800,000 – 899,999

10. 900,000-999.999

11. Over 1.000.000

88. Do not know

99. Do no wish to disclose

Question 4

What basic education/schooling do you have? *(only one tick)*

1. 7 years

2. 8 years

3. 9 years, middle-school exam

4. 10 years, GCSE

5. A-levels

6. Diploma in commerce or diploma in technology

7. Other

Question 5

What sort of vocational training or further education do you have besides level of schooling? *(only one tick)*

1. Skilled worker

2. Basic vocational education (but not the second part)

3. Apprenticeship or basic vocational education

4. Other vocational training of at least one year’s duration

5. Further education (less than 3 years)

6. Further education (3-4 years)

7. Further education (more than 4 years)

8. Other type of vocational training

9. No vocational training

Question 6

What is your legal marital status?

1. Married – go to question Text 2

2. Widower – go to question 7

3. Divorced – go to question 7

4. Separated – go to question 7

5. Unmarried – go to question 7

Question 7

Are you cohabitating?

1. Yes

2. No

| Text 2:  In the next questions I would like to ask you to image that you are in your doctor’s office. First the doctor gives you some information which you will then have to consider.    You have to imagine that your doctor tells you that you have raised cholesterol levels and thus an increased risk of a coronary thrombosis. This means that within the next 10 years 15 out of 100 persons with a risk like yours, i.e. 15%, will have a fatal coronary thrombosis.  To get an idea how great the risk of dying of a coronary thrombosis is, the figure on this card [Show card 1] compares the risk of having a thrombosis with other risks. The figures in the columns show a person’s risk of dying of cancer or a car accident within the next 10 years.  As it happens your doctor can offer you a drug as preventive treatment against having a coronary thrombosis. The medicine should be taken once a day. There may be side-effects of the medicine like for instance insomnia, headache, rashes or digestive problems. The side-effects can be permanent or temporary. You have to go for a check-up at your doctor’s every 6 months, and the cost of the medicine is approx. DKK 500 per year.  (Show card 1A)  If 100 persons with raised cholesterol levels like you take the medicine, 5 more will be alive after 10 years than if they did not take the medicine. |
| --- |

Question 8

Having been given this information, would you start on the medication offered?

1. Yes

2. No

8. Don’t know

Question 9

If instead you had to give your answer on a scale, to what degree would you wish to take the medication? You can reply by means of this card [Show card 2], where 0 means “not at all” and 10 means “to a very high degree”.

| Text 3:  I am now going to tell you a bit more about the same medicine, which prevents coronary thrombosis. I am going to tell you about the effect of the medicine in different ways. Then you will again have the opportunity to say whether you would take the medicine.    The effect of the medicine can actually also be explained by means of this picture [Show Card 3]. If individuals with raised cholesterol levels do not take the medicine, 15 out of 100 persons will die of a coronary thrombosis within the next 10 year. If individuals, on the other hand, take the medicine, 10 out of 100 will die of a coronary thrombosis within the next 10 years.  This can also be expressed in another way [Show Card 4 and read aloud]:  If 20 persons with raised cholesterol levels like you take the medicine, one more will be alive after 10 years than if they did not take the medicine.  If a person with raised cholesterol levels like you takes the medicine, the risk of dying of a coronary thrombosis will be reduced by 33% within the next 10 years.    If persons with raised cholesterol levels like you take the medicine, they will on average live 8 months longer than if they did not take the medicine. |
| --- |

*Question 10*

Would you having received this further information wish to take the medicine?

1. Yes

2. No

8. Don’t know

*Question 11*

If instead you had to give your answer on a scale, to what degree would you wish to take the medication? You can reply by means of this card [Show Card 5], where 0 means “not at all” and 10 means “to a very high degree”.

Finally I would like to ask you a few follow-up questions

Question 12

Which one of these four ways of giving information did you like best? (Show both Card 3 and Card 4)

1. Code 1 on Card 4 (persons)

2. Code 2 on Card 4 (percentages)

3. Code 3 on Card 4 (number of months life is prolonged)

4. Code 3 (figure with faces)

Question 13

If you think about the information you have just said that you liked best, how easy or difficult did you find understanding the information?

1. Very easy

2. Fairly easy

3. Fairly difficult

4. Very difficult

8. Don’t know

FILTER:

If question 10 = code 1 go to question 14

If question 10 = code 2 go to question 15

Question 14

What did you consider most important when you assumed you would like to accept the medicine? Please choose one of the answers listed on this card (Show Card 7) *(Interviewer.:only 1 reply should be given).*

1. Considerations for my health

2. I trust that the doctor knows what is best for me

3. That it is my responsibility towards my family to stay healthy

4. Anything else *(interviewer.: make a note*____________________________________

8. Don’t know

Question 15

What did you consider most important when you assumed that you would not accept the medicine? Please choose one of the answers listed on this card (Show Card 8) *(Interviewer.:Only 1 reply should be given)*

1. I think that the effect of the medicine is too small

2. I wish to avoid side-effects of medicine

3. I do not wish to have the extra expense

4. I do not like taking medicine

5. I found it hard to understand the information I received

6. I do not believe that the medicine works

7. It is better to change my lifestyle than to take medicine

8. Anything else *(interview.:make a note)*______________________________________

88. Don’t know

Question 16

Have you ever had raised cholesterol levels measured?

1. Yes

2. No

8. Don’t know

Question 17

Have you ever had raised blood pressure measured?

1. Yes

2. No

8. Don’t know

Question 18

Have you ever had a cerebral thrombosis?

1. Yes

2. No

8. Don’t know

Question 19

Have you ever had a coronary thrombosis or any other heart disease?

1. Yes

2. No

8. Don’t know

Question 20

Have any of your close family members (father, mother, brother, sister) ever had a coronary thrombosis?

1. Yes

2. No

8. Don’t know

Question 21

Have any of your close family members (father, mother, brother, sister) ever had a cerebral thrombosis?

1. Yes

2. No

8. Don’t know

Question 22

Image that you throw a coin up in the air. How many times on average do you think it will be ‘heads’, if the coin is tossed 1000 times?

___________ times out of 1000

Question 23

Image that you roll an ordinary dice 1000 times. Out of 1000 rolls how many times do you think you will roll an even number, i.e. 2, 4 or 6?

_____ times out of 1000

Question 24

If the probability of getting a disease is 10%, how many persons out of 100 would be expected to get the disease?

_____ persons out of 100

Question 25

Imagine that you want to buy a sweater, which is reduced by 25% of the original price of DKK 400. How much would you have to pay for the sweater? *(Interviewer: read all the response options aloud)*

1. DKK 100

2. DKK 250

3. DKK 300

4. DKK 400

5. DKK 500

Thank you very much for your help!

**Questions for the interviewer to answer after the interview:**

To what degree do you estimate that the informant understood the full information given in text 3?

1. The informant seemed to find it very easy to understand the information

2. The informant seemed to find it fairly easy to understand the information

3. The informant seemed to find it fairly difficult to understand the information

4. The informant seemed to find it very difficult to understand the information
